# Gradient wettability induced by deterministically patterned nanostructures

**DOI:** 10.1038/s41378-020-00215-0

**Published:** 2020-11-30

**Authors:** Siyi Min, Shijie Li, Zhouyang Zhu, Wei Li, Xin Tang, Chuwei Liang, Liqiu Wang, Xing Cheng, Wen-Di Li

**Affiliations:** 1 Department of Mechanical Engineering, The University of Hong Kong, Hong Kong, 999077 China; 2 Department of Materials Science and Engineering, Southern University of Science and Technology, Shenzhen, 518052 China; 3 HKU-Zhejiang Institute of Research and Innovation (HKU-ZIRI), Hangzhou, 311305 Zhejiang China

**Keywords:** Structural properties, Structural properties

## Abstract

We report a large-scale surface with continuously varying wettability induced by ordered gradient nanostructures. The gradient pattern is generated from nonuniform interference lithography by utilizing the Gaussian-shaped intensity distribution of two coherent laser beams. We also develop a facile fabrication method to directly transfer a photoresist pattern into an ultraviolet (UV)-cured high-strength replication molding material, which eliminates the need for high-cost reactive ion etching and e-beam evaporation during the mold fabrication process. This facile mold is then used for the reproducible production of surfaces with gradient wettability using thermal-nanoimprint lithography (NIL). In addition, the wetting behavior of water droplets on the surface with the gradient nanostructures and therefore gradient wettability is investigated. A hybrid wetting model is proposed and theoretically captures the contact angle measurement results, shedding light on the wetting behavior of a liquid on structures patterned at the nanoscale.

## A new method for producing wettability gradients

A new approach developed by researchers in China makes it possible to manufacture materials with continuously varying wettability along their surface. A team led by Wen-Di Li of the University of Hong Kong and Xing Cheng of Southern University of Science and Technology came up with an approach that begins with interference lithography to create a variable intensity pattern with circular symmetry. This created nanostructures in a photoresist, which then served as the base for fabricating a UV-cured mold, which could then be used to transfer the pattern onto a polymer. This approach eliminates the need for costly fabrication processes. The team used this approach to generate a surface with a wettability gradient and investigate its properties, which they found were best described by a hybrid combination of the classic Wenzel and Cassie wetting models.

## Introduction

Surfaces with spatially varied wettability are essential for numerous interfacial applications. A typical example is the realization of droplet self-propulsion^1^ due to a passive driving force originating from an imbalanced surface tension, which could be further integrated into microfluidic devices^2^ to achieve controlled liquid flow. Surfaces with gradient wettability are also applied in thermal management^3,4^ and chemical sensors^5^. Many biological processes, such as cell adhesion, migration, and proliferation, are also guided by wettability gradients^6^. In addition, surfaces with gradient wettability can act as a high-throughput analysis tool for systematic studies that need continuously varying wettability within a single experiment^7^.

To date, surfaces with gradient wettability have been realized through several types of chemical gradients. For instance, surfaces are modified with hydrophobic and hydrophilic coatings with gradient coverage ratios to produce gradient wettability^1^, volatile chemicals are diffused over a distance to create a spatially varied vapor concentration and react with the substrate^8,9^, and a surface is gradually immersed into a modifying liquid to generate a treatment time gradient across the length of the substrate^10,11^. Alternatively, many researchers have devoted attention to the fabrication of surfaces with gradient wettability arising from morphological gradients, as micro/nanostructures play an equally significant role in surface wettability. Laser texturing^5,12^ and photolithography^13–17^ have been utilized to generate patterns with gradient feature dimensions. Another widely used method is to employ particles^18–25^ that progressively vary either in number density or particle size on the target surface. For instance, Yang and colleagues^24^ utilized the self-assembly of microspheres to form a uniform array on a substrate, followed by inclined reactive ion etching to generate a geometric gradient. Bhat et al.^19,20^ used a pre-existing chemical gradient to generate a surface with spatially varied nanoparticle coverage. In addition, electrochemistry has also been applied to fabricate morphology gradients, where in-plane potential gradients are utilized to generate progressively varied nanopores^26^. Many other approaches based on magnetic^27^, thermal^28–30^, and humidity^31^ gradients have also been used to fabricate gradient topography surfaces.

Although many methods have been developed to generate a spatially varied wettability, precise and deterministic fabrication is still a challenge. Chemical gradient-based surfaces have limitations in terms of their resolution, as high-precision control of both the spatial position and chemical parameters is challenging. The alternative approach based on topography gradients has the potential to overcome this limitation. To date, most morphology-induced gradient wettability involves structures with feature sizes ranging from a few to tens of micrometers that take advantage of easily accessible patterning approaches. However, surfaces with gradient nanostructures are of great interest, as they can not only produce gradient wettability on micrometer length scales to benefit microdroplet manipulation but also create a steeper gradient that could generate a larger driving force for droplets^32,33^ and increase the thermal management efficiency^3^. The vast majority of the reported methods for fabricating gradient nanostructures have limitations regarding determinacy and reproducibility, which affect their potential to be applied in practical mass manufacturing processes.

Here we employed interference lithography to fabricate a surface with gradient wettability generated from deterministic and ordered large-area gradient nanostructures. Conventional interference lithography emphasizes pattern uniformity^34,35^; therefore, a uniform exposure intensity over the patterning area is desired. To fabricate spatially varying structures, we intentionally utilized the nonuniform Gaussian-shaped intensity distribution of the two coherent beams to realize spatially varying patterns with circular symmetry. In addition, we developed a facile fabrication method to directly transfer the photoresist pattern generated from interference lithography into an ultraviolet (UV)-cured high-strength replication molding material, which eliminates high-cost reactive ion etching and e-beam evaporation processes in the mold fabrication process. This facile mold was then applied for the reproducible production of polymer films with gradient wettability using thermal-nanoimprint lithography (NIL). Moreover, we used this gradient wettability surface to study the wetting behavior on the nanostructures, which cannot be fully described by the classic Wenzel or Cassie models^36–40^. The theoretical calculation results from the hybrid model effectively captured the experimental measurements of apparent contact angles, shedding light on the diverse immersion situations of water droplets on surfaces patterned with various nanostructures.

## Results and discussion

### Interference lithography for fabricating concentric gradient nanostructures

**Fig. 1:**
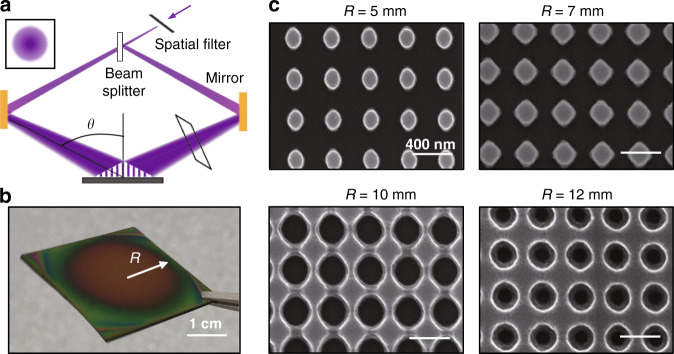
Fabrication of gradient nanostructure by interference lithography. **a** Schematic of the interference lithography principle and laser source with a Gaussian distribution for gradient pattern formation. **b** Photograph of the photoresist pattern after development with two orthogonal interference exposures during lithography, where *R* is the distance from the center. **c** SEM images of photoresist nanostructures at different positions along the radial direction

Interference lithography is a kind of maskless photolithography that can generate regular arrays of submicron structures. In our experiment, interference lithography is applied to deterministically fabricate large-area concentric gradient nanostructures by utilizing the Gaussian-distributed light intensity profile of the laser source. During the interference lithography process, two coherent beams overlap and produce interference at the target substrate, as depicted in Fig. 1a. The intensity profile of each laser beam decreases from the center to the edge, as schematically shown in the inset. Thus, the resultant interference intensity varies at each position, which is determined not only by the phase difference but also by the spatially varying light intensity.

A positive-tone photoresist is applied to record the intensity profile of two orthogonal interference exposures during lithography. Figure 1b shows a photograph of the structured photoresist pattern after development. The nanostructures are distributed in a round region that comes from the round-shaped contour lines of the Gaussian beam. The pattern is nearly concentrically distributed, and the morphology is characterized by scanning electron microscope (SEM) along the radial direction, as indicated by the black arrow. The SEM image shows nanopillars at the center where the Gaussian beam has the maximum intensity. The diameter of the nanopillars increases with distance from the center. Nearby pillars start to merge, thus transforming into a hole array, and the diameter of the holes decreases as the distance from the center increases (Fig. 1c). The gradient of the nanostructure profile is also in accordance with the theoretical calculation and modeling of the intensity distribution of the interference pattern (Supplementary Notes 1 and 2). Although the geometric shape and size of the nanostructures are spatially varied, the period remains constant at 370 nm, as it is solely dependent on the angle between the two laser beams.

### Transferring gradient nanostructures into a polymer film to fabricate a surface with gradient wettability

**Fig. 2:**
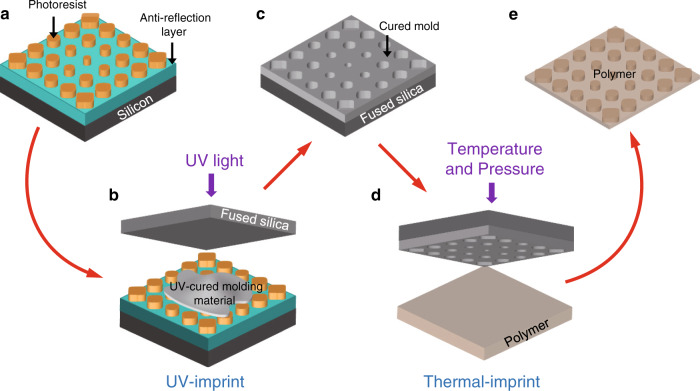
Schematic illustration of the pattern transfer process. **a** Formation of concentric gradient nanostructures in the photoresist by interference lithography. **b** Replication of the photoresist pattern into a UV-cured molding material on a fused silica substrate by UV-NIL. **c** Demolding of the cured mold from the photoresist pattern. **d** Transfer of the pattern into a polymer by thermal-NIL. **e** Separation of the patterned polymer film from the mold

A surface with gradient wettability is fabricated by transferring the photoresist pattern into a functional polymer. Cyclic olefin copolymer (COC) is chosen here because of its unique combination of many promising properties (Supplementary Note 4), such as low water absorption, low surface energy, high strength, flexibility and high chemical resistance to acids and alkalis, that make it an ideal material for many applications^41^. The fabrication process is schematically depicted in Fig. 2a–e. After interference lithography, concentric gradient nanostructures are formed on the photoresist, which gradually change from pillars to holes from the center to the edge (Fig. 2a). Then, a UV-cured molding material is applied to replicate the photoresist structure by UV-NIL (Fig. 2b). After demolding from the photoresist pattern, the cured material can be utilized as a thermal-NIL template (Fig. 2c). Finally, thermal-NIL (Fig. 2d) is applied to transfer the concentric gradient structures from the template to COC films (Fig. 2e).

**Fig. 3:**
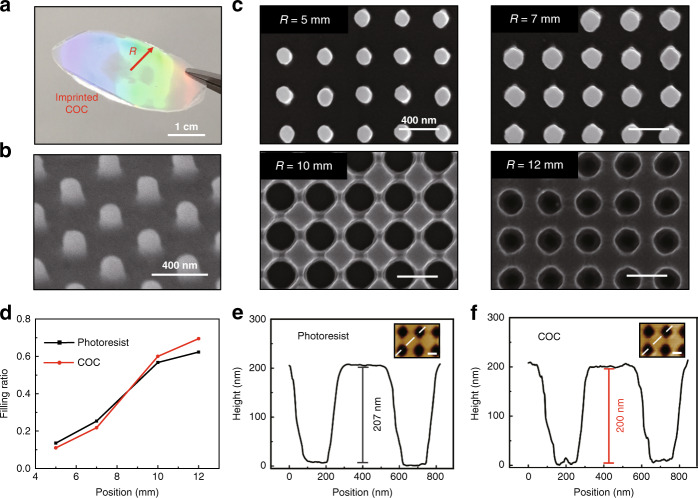
Evaluation of pattern transfer fidelity. **a** Imprinted COC film. **b** SEM image of COC nanopillars viewed with a tilting angle of 45°. **c** Top-view SEM images of representative structures on the imprinted COC film at different positions. **d** Filling ratios of the photoresist pattern and COC film at the corresponding positions. **e**, **f** AFM height images and cross-sections of nanoholes on the photoresist and imprinted COC film. The white dashed lines in the AFM images mark the positions of the cross-sections. The scale bar is 200 nm

To investigate the structural fidelity during the whole transfer process, the morphology of the photoresist pattern (Fig. 1b) and replicated COC film (Fig. 3a) are analyzed at corresponding positions by SEM. As indicated by these SEM images, the COC copy carries almost the same morphology as the original resist. The filling ratio, which is the ratio between the area occupied by the protruding region and the total area of the unit cell, is quantitatively measured at the corresponding positions of the photoresist and COC sample (Fig. 3d). The differences in filling ratio between the original resist and replicated films are <8%. In addition, atomic force microscope (AFM) is also applied to characterize the heights of the photoresist pattern and the replicated COC film (Fig. 3e, f). The difference in height is <10 nm. Thus, consistency in the structural dimensions of the original photoresist pattern and replicated COC films at different positions along the radial direction of the gradient pattern can be confirmed. In addition to COC films, other thermoplastic polymers with a relatively low transition temperature (<200 °C) are also suitable for this process. PET films with concentric gradient nanostructures are fabricated through this process as a demonstration. The morphology and height of the replicated PET film are analyzed by SEM and AFM (Supplementary Note 3). The results further show the high consistency of the transfer process. However, it should be noted that this transferring process is mainly suitable for fabricating structures with non-re-entrant profiles in materials that are not easily deformed.

### Varying contact angle on the patterned COC film

**Fig. 4:**
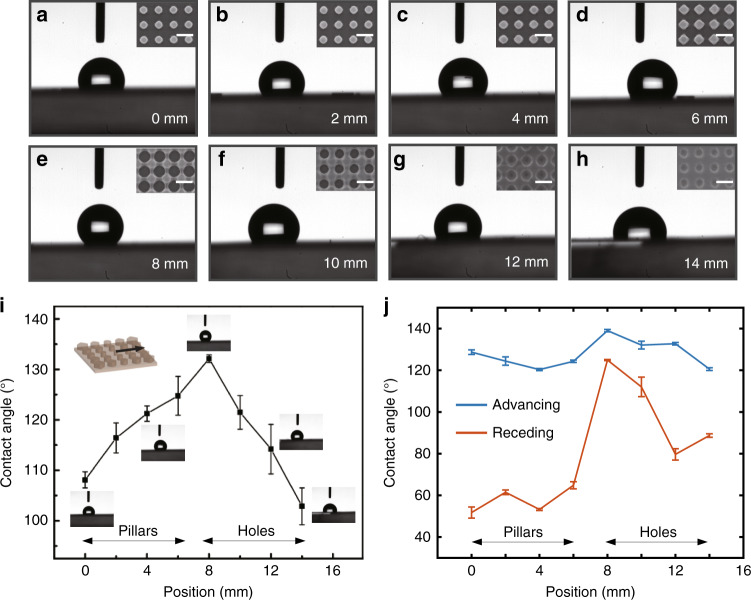
Contact angles on gradient nanostructures. **a**–**h** Optical images of water droplets at different positions of the concentric gradient COC pattern (0 ~ 14 mm from the center); the insets are SEM images of the corresponding nanostructures, and the scale bar is 400 nm. **i** Apparent contact angle at corresponding positions of **a**–**h**; the error bars indicate SDs from at least four measurements. **j** Advancing and receding contact angles at corresponding positions in **a**–**h**

The unique wetting features on a surface with gradient wettability can be revealed by contact angle measurements. The static sessile drop method is applied to characterize the relationship between the apparent contact angle and the position on the patterned COC film. The droplet volume was 3.5 µL. The changing droplet shape leads us to conclude that as the geometric parameter continuously changes from the pattern center to the edge, the COC film becomes increasingly hydrophobic, with the hydrophobicity peaking at the *R* = 8 mm position (Fig. 4a–e); then, the hydrophobicity decreases from *R* = 8 mm to *R* = 14 mm (Fig. 4f–h). During water droplet deposition, the droplet stays centered below the needle at *R* = 0, 8, and 14 mm (Fig. 4a, e, h). In contrast, jumps occur when the droplet initially contacts the patterned COC film at other positions (*R* = 2, 4, 6, 10, and 12 mm). This phenomenon has been observed on surfaces with gradient wettability^42^. The jumping process is visualized in Supplementary Fig. S5. The droplet jumps to the right side of the needle position in the region from *R* = 2 mm to *R* = 6 mm (Fig. 4b–d), whereas it jumps to the left from *R* = 10 mm to *R* = 12 mm (Fig. 4f, g). The relative locations of the droplet and needle can verify the changing hydrophobicity on the COC film. Water droplets at *R* = 0, 8, and 14 mm have balanced capillary forces on the left and right sides of the droplets. These three positions correspond to the geometric center of the sample (*R* = 0 mm), the maximum contact angle (*R* = 8 mm), and the edge of the sample with small holes (*R* = 14 mm). However, the hydrophobicity of the patterned COC film monotonically changes between *R* = 2 mm and *R* = 6 mm or between *R* = 10 mm and *R* = 12 mm. This causes an unbalanced surface tension and, thus, the droplet jumps upon contact with the patterned COC film. The opposite jump directions of these two spatial ranges signify the opposite changes in the hydrophobicity. This effect is unique to gradient substrates since it is not observed for flat surfaces or surfaces with uniform micro/nanostructures.

The quantitative results for the apparent water contact angles at various positions along the radial direction of the patterned COC film are shown in Fig. 4i. Due to the small droplet volume, the space between the left and right triple-phase lines is too small to generate an obvious difference between the left and right contact angles; thus, the mean apparent contact angle of the droplet is used here^24^. The intrinsic contact angle *θ*_*e*_ of the COC sample is 99.1° ± 1.5°. As the position moves from the center, the contact angle gradually increases from 108.1° to 132.1° from the center to *R* = 8 mm with gradually increasing pillar diameter and then decreases to 102.9° at *R* = 14 mm due to the increasing filling ratio, which also indicates a decrease in the hole diameter. The contact angle peaks at *R* = 8 mm, which is the transition region from pillars to holes. The water contact angle is determined by the water–air and water–COC contact areas, which provides an indication of the diverse immersion situations of water droplets on ordered nanostructures with different duty ratios. In addition to apparent contact angles, advancing and receding contact angles are also characterized. In the measurement, a sessile drop is expanded from 3.5 to 13.5 μL and then contracted back to 0.5 μL at a rate of 1 μL/s. On the flat COC surface, the advancing angle is 107.2° ± 0.8°, whereas the receding angle is 84.3° ± 0.4°. On the nanostructured COC film, the results at various positions are shown in Fig. 4j. The advancing angle varies between 120° and 140° on the gradient COC nanostructures. The advancing angle is not significantly affected by the shape and size of the nanotexture, as the advancing front just lays atop nanostructures. However, the receding line is pinned by the structures, a scenario where the nanoscale topography plays a key role. As a result, the receding angle shows an obvious variation.

### Wetting models for a nanostructured surface with gradient wettability

The structured COC film is utilized to study the diverse immersion situations for water droplets on ordered nanostructures with different duty ratios. If a liquid droplet reaches a thermodynamically stable state on a solid surface, the apparent contact angle θ^*^ is determined by the liquid–vapor interface area (*A*_LV_), liquid–solid interface area (*A*_LS_), projection area (*A*_P_), and intrinsic contact angle *θ*_e_. The thermodynamic stability of droplets on COC nanostructures is verified (Fig. S4), if *f*_1_ = *A*_LS_/*A*_P_ and *f*_2_ = *A*_LV_/*A*_P_, as given by the Cassie–Baxter relation:^43,44^

1 $$\begin{array}{*{20}{c}} {\cos \theta ^ \ast = f_1\cos \theta _{\mathrm{e}} - f_2} \end{array}$$

**Fig. 5:**
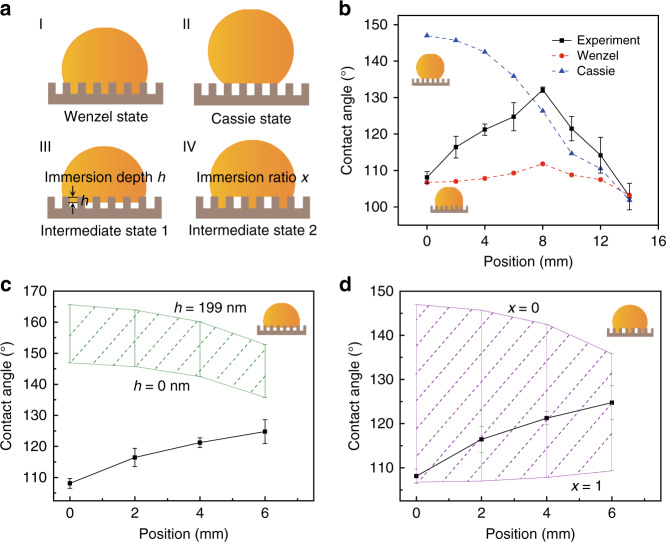
Possible models of water wetting behavior changes over gradient nanostructures. **a** Schematic illustration of two classic wetting models, namely, Wenzel and Cassie, and two intermediate state models, where one has immersion depth *h* as the variable parameter, and the other has immersion ratio *x* as the variable parameter. **b** Contact angle at different positions of the COC film with a concentric gradient structure from the experiment and theoretical calculation of the Wenzel model and the Cassie model. **c** Comparison of the experimental contact angle and theoretically calculated possible range (the area with green dashed lines) of the intermediate state 1 model at each position from *h* = 0 nm to *h* = 199 nm (where the structure depth is 200 nm). **d** Comparison of the experimental contact angle and theoretically calculated possible range (the area with purple dashed lines) of the intermediate state 2 model at each position from *x* = 0 (where no groove is immersed in water) to *x* = 1 (where all grooves are immersed in water)

Therefore, this equation is applicable on a nanostructured COC surface. First, two classic wetting models, the Wenzel model and the Cassie model, are applied. The Wenzel model describes the state in which water thoroughly fills the nanostructures, as depicted in Fig. 5a-I. In this situation, *f*_1_ = *r* and *f*_2_ = 0, and the equation turns into the Wenzel equation: cosθ^*^ = *r*cos*θ*_e_, where *r* is the roughness of the structured surface. The Cassie model describes another possible wetting behavior in which a water droplet stands on the nanostructure and air is trapped in the trenches, as shown in Fig. 5a-II. In this situation, *f*_1_ = *f*_s_ and *f*_2_ = 1 − *f*_s_ and the equation turns into the Cassie equation: cosθ^*^ = *f*_s_(1 + cos*θ*_e_) − 1. To simplify the calculation, the nanostructures are assumed to be standard pillars and holes with ideally vertical sidewalls. Details of all the calculations can be found in the Supplementary Note 7. The theoretical contact angles of the Wenzel and Cassie models are plotted in red and blue curves in Fig. 5b. The Wenzel state calculation result exhibits the same trend as the experimental results but shows an increasing difference with increasing contact angle. As the Wenzel ratio *r* does not exceed 1.92, the equation predicts a variation range of only 8.4°, which is significantly lower than the experimental results. The Cassie state calculation result, as shown in Fig. 5b, matches the experimental results from *R* = 8 mm to *R* = 14 mm; thus, the water droplet wetting behavior can be described with the Cassie state. This result is intuitive since the hydrophobic pores in the nanohole region are too small to be penetrated by water droplets at atmospheric pressure. However, from *R* = 0 mm to *R* = 8 mm, the theoretical result from the Cassie equation significantly deviates from the experimental results both in value and tendency, so this situation clearly does not hold in the nanopillar region.

To identify the wetting state, the breakthrough pressure is quantitatively evaluated at various positions of the structured COC film (Supplementary Note 8). Along the radial direction, the breakthrough pressure gradually increases from 58.5 to 94.2 kPa in the pillar region (*R* = 0 to 6 mm) and from 150.2 to 450.5 kPa in the hole region (*R* = 8 to 14 mm). It should be noted that underneath a droplet, the air entrapped between pillars is connected to the atmosphere, while the air inside the holes is isolated. As a result, the actual breakthrough pressure of nanoholes may be higher than the calculated value due to the compression of the underlying air pocket. Therefore, nanoholes in the COC are difficult to penetrate and droplets remain in the Cassie state, whereas for nanopillars the breakthrough pressure is relatively low, so droplets may penetrate the structures during a disturbance. Due to benefits from the compartmentalization of the underlying air pocket, the hole surface is considered superior to the pillar surface in many scenarios, especially in preventing catastrophic wetting transitions^45,46^. Here, the wetting state in the nanopillar region is assumed to consist of mixed domains of the Wenzel state, which have intimate solid–liquid contact, and the Cassie state, where small air pockets are trapped between the solid and liquid. Two possible hybrid models are proposed for the nanopillar region. The first intermediate state describes the situation in which a droplet fills all the grooves to equal depth *h*, as depicted in Fig. 5a-III. The second intermediate state describes the situation in which the droplet is suspended on some grooves, whereas the other grooves are fully filled with immersion ratio *x*, as depicted in Fig. 5a-IV.

For intermediate state 1, the immersion depth is the critical parameter, which varies from 0 to 199 nm (the depth of the holes is 200 nm). We calculated the possible contact angle range for two extreme cases (*h* = 0 nm and *h* = 199 nm) in the COC nanopillar region. The range is shown in the green shadowed region in Fig. 5c. If the droplet immerses into the nanostructures, the theoretical contact angle is larger than that under the Cassie state. As a result, the theoretical range obviously has no overlap with the experimental results. Therefore, the water wetting behavior on COC nanopillars cannot be described by intermediate state 1.

For intermediate state 2, the groove immersion ratio is the critical parameter, which varies from 0 (fully nonwetting) to 1 (fully wetting); these two extreme cases correspond to the Cassie state and the Wenzel state, respectively. The apparent contact angle of intermediate state 2 is the weighted average of these two states. The range of the theoretical achievable contact angle in the COC nanopillar region is shown in the purple shadowed part in Fig. 5d. The measured apparent contact angles fall in this domain; thus, in the COC nanopillar region, the water wetting behavior can be modeled using an intermediate state in which the droplet is suspended on some grooves, while the other grooves are fully filled. The hypothesis is verified using a 0.1 g/mL NaCl solution. The droplets of the NaCl solution are dropped to cover the whole structured COC film. After 5 min, the liquid is removed at a rate of ~3 μL/s. The COC surface is then analyzed by SEM. NaCl solute can be found in some parts of the nanopillar region, which provides an indication for the partial filling hypothesis (Supplementary Fig. S10). Furthermore, in the region from *R* = 0 mm to *R* = 6 mm, the immersion ratio *x* gradually decreases from 1 to 0.4. This is reasonable since with increasing pillar diameter, the breakthrough pressure increases and, therefore, the water immersion ratio decreases accordingly.

## Conclusion

In summary, we successfully fabricate a large-scale nanostructured surface with continuously varying wettability. The novel fabrication method combines nonuniform interference lithography, UV-NIL, and thermal-NIL to deterministically fabricate gradient nanostructures on polymer films. In the processes, a facile UV-cured template is introduced and exhibits good transfer fidelity from photoresist patterns to COC nanostructures. Nanostructure-induced gradient wettability has the potential to manipulate microdroplets and create a steeper gradient. In addition, the diverse wetting behaviors of water droplets on nanostructures are investigated. A hybrid wetting model is proposed and theoretically captures the contact angle measurement results in the nanopillar region. It describes the wetting state in which a droplet is suspended on some grooves while the other grooves are fully filled. The immersion ratio is dependent on the duty ratio of the COC nanopillars. This study sheds light on diverse immersion situations of water droplets on surfaces patterned with various nanostructures.

## Materials and methods

### Fabrication of concentric gradient nanostructures by interference lithography

Silicon wafers (Suzhou Crystal Silicon Electronic & Technology Co., Ltd, China) were cut into 3.5 × 3.5 cm pieces, treated with an oxygen plasma (Potentlube, China) for 2 min, cleaned with a cotton swab and acetone, and finally thoroughly rinsed with acetone. Then, the silicon substrates were further cleaned by ultrasonication in isopropanol for 1 min before being dried with nitrogen. AZ Barli II-200 (MicroChemicals GmbH, Germany) was spin-coated at 2000 r.p.m. for 60 s and then baked on a hotplate at 200 °C for 2 min to produce an anti-reflection layer. AZ MiR 701 (MicroChemicals GmbH, Germany) photoresist was diluted to twice the original volume with 2-(1-methoxy) propyl acetate (Acros Organics, Belgium) and spin-coated on top of the ARC layer at 5000 r.p.m. for 60 s, followed by soft baking at 90 °C for 1 min. A home-built two-beam fiber-optic interference lithography system was used for the fabrication of gradient nanostructures. A laser source with a 405 nm operating wavelength was applied in this system. The laser was split into two sub-beams by a beam splitter and coupled into two polarization-maintaining single-mold fibers. The mode field diameter of the fibers was 3.3 µm. When the laser beam was emitted from the single-mode fiber facet, it maintained a Gaussian intensity profile. The photoresist was exposed to a 10 mJ cm^−2^ exposure dose (center dose). The expansion distances of the two laser beams were 20 cm. The angle between the two laser beams was set at 33.18° to fabricate a pattern with a 370 nm period. The photoresist was then developed for 1 min in a mixture of AZ 351B and deionized (DI) water with a volume ratio of 1 : 4. The samples were finally rinsed in DI water and blow-dried with compressed air. The characterization of the photoresist patterns was performed using a LEO 1530 SEM and Bruker MultiMode-8 AFM.

### Fabrication of UV-curable template

Fused silica substrates were treated by an oxygen plasma for 2 min and then cleaned. Prime (micro resist technology GmbH, Germany) was spin-coated at 4000 r.p.m. for 60 s and then baked on a hotplate at 150 °C for 5 min as an adhesive layer. The patterned photoresist samples were treated by oxygen plasma for 5 s, and a perfluorodecyltrichlorosilane self-assembled monolayer coating was then formed on the photoresist by a conventional vapor phase deposition process operated at 120 °C for 15 min. Then, 0.065 g Ormostamp liquid (Micro Resist Technology GmbH, Germany) was dropped on the photoresist sample. The sample surface was then gently covered with the Prime-coated fused silica and left to stand for ~15 min to ensure the complete spread of Ormostamp on the photoresist sample surface. Then, 365 nm UV light from a photolithography machine (URE 2000/35, Chinese Academy of Sciences, China) was applied to cure the Ormostamp with a dose of 7.3 J cm^−2^. Afterward, the cured mold was separated from the photoresist sample and thoroughly rinsed with acetone and isopropanol.

### Fabrication of COC films with concentric gradient nanostructures

The nanostructures were transferred from the Ormostamp template to a 100 µm thick COC film (Grade 8007, TOPAS, Germany) by thermal-NIL using a home-built apparatus consisting of electrically heated platens with a temperature controller (Specac Ltd, UK), a hydraulic press (Specac Ltd, UK), and a chiller (Grant Instruments, UK). During the thermal-NIL process, the platens were heated to 120 °C and an imprinting pressure of 3.2 MPa was applied and held for 5 min. The heated platens were then cooled to the demolding temperature of 40 °C. Finally, the COC film was peeled from the Ormostamp template. The characterization of the samples was performed using a LEO 1530 SEM and a Bruker MultiMode-8 AFM.

### Water contact angle studies

The water contact angles were measured by using Model 100SB (Sindatek Instruments Co., Ltd, Taiwan). For the apparent contact angle measurements, water droplets with a quantity of 3.5 µL from a fixed needle were gently deposited on the sample surfaces at room temperature. During the advancing and receding angle measurements, a sessile drop expanded from 3.5 to 13.5 μL and then contracted back to 0.5 μL at a rate of 1 μL/s on the sample surface. The contact angles were determined with Model 100SB software. The sample stage was precisely moved in the *x* and *y* directions, enabling position control of the gradient sample.

## Supplementary information

Supplementary Information
