# Supplementary material for: Gradient wettability induced by deterministically patterned nanostructures
Source: Microsyst Nanoeng. 2020 Nov 30;6:106. doi: 10.1038/s41378-020-00215-0 (PMC8433471; doi:10.1038/s41378-020-00215-0)
Supplement: Supplementary file 1 — Supplementary Information [file 41378_2020_215_MOESM1_ESM.docx]

**Supplementary Information**

**Gradient Wettability Induced by Deterministically Patterned Nanostructures**

Siyi Min^1,2^, Shijie Li^1^, Zhouyang Zhu^1^, Wei Li^1^, Xin Tang^1^, Chuwei Liang^1^, Liqiu Wang^1,3^, Xing Cheng^2*^, and Wen-Di Li^1,3*^

^1^*Department of Mechanical Engineering and, The University of Hong Kong, Pokfulam, Hong Kong, 999077, China*

^2^*Department of Materials Science and Engineering, Southern University of Science and Technology, Shenzhen, 518052, China*

^3^*HKU-Zhejiang Institute of Research and Innovation (HKU-ZIRI), Hangzhou, 311305, Zhejiang, China*

E-mail: [chengx@sustech.edu.cn](mailto:chengx@sustech.edu.cn) (XC) and [liwd@hku.hk](mailto:liwd@hku.hk) (WDL)

**Supplementary Information**

**Note 1.** **Theoretical calculation of intensity distributions**

The intensity profile of the laser beams is Gaussian-shaped and is determined by the mold field diameter $w_{0}$ of the fiber, given by

$$I_{\mathrm{beam}}=\frac{I_{0}}{1+\left( \frac{\lambda z}{\pi w_{0}^{2}} \right)^{2}}\exp\left( -\frac{2r^{2}}{w_{0}^{2}\left( 1+\frac{\lambda z}{\pi w_{0}^{2}} \right)^{2}} \right)$$

where $\lambda$ is laser operating wavelength, $I_{\mathbf{0}}$ is the laser intensity at the center of fiber facet, $r$ is the radial distance from the central axis of the beam, $z$ is the beam expansion distance.

The overlap of these two coherent laser beams results in interference, the intensity distribution of the interference pattern is as follows:

$$I={2I}_{\mathrm{beam}}+2I_{\mathrm{beam}}\cos\left( \frac{2\pi x}{\Lambda} \right)$$

$$\Lambda=\frac{\lambda}{2sin\theta}$$

where $x$ is the coordinates of the positions on the sample stage, $\Lambda$ is the period of the interference pattern.

**Note 2. Modeling of intensity distributions and corresponding photoresist profiles**


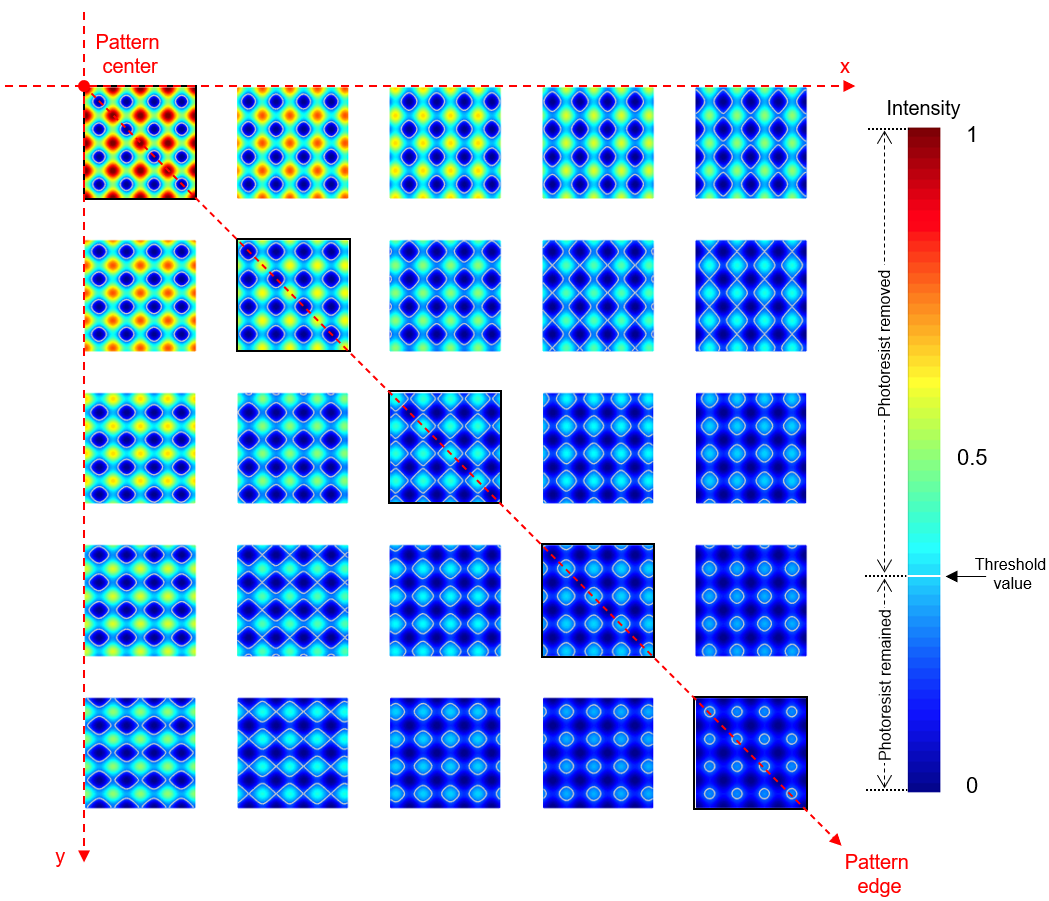


**Fig. S1:** Modeling of intensity distributions in a quarter region of the interference pattern, which comes from two orthogonal non-uniform exposures. The white envelope corresponds to a threshold value of the exposure; therefore, it can also be regarded as photoresist profiles.

**Note 3. Morphology of imprinted PET film**


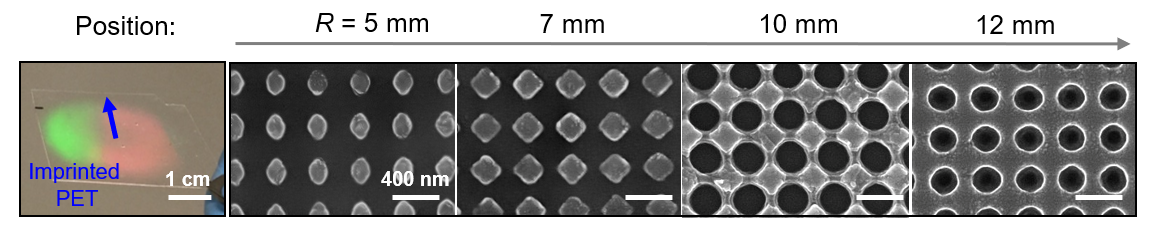


**Fig. S2:** Imprinted PET film and its SEM images at different positions as shown by the arrow and position label.


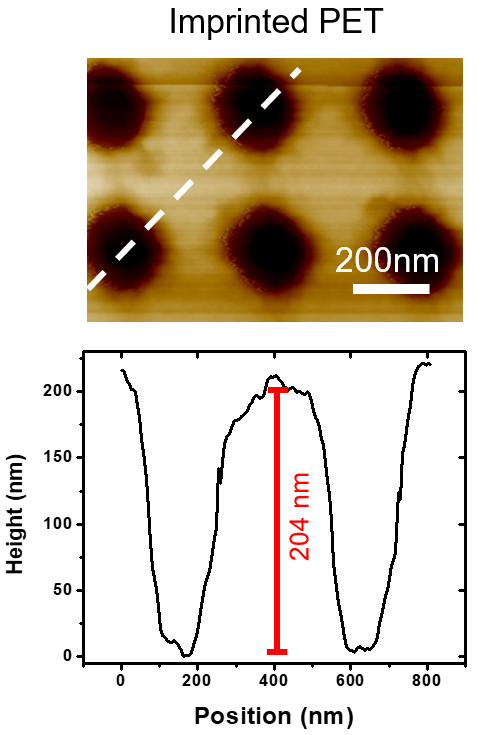


**Fig. S3:** AFM height image and cross section of nanoholes on imprinted PET film. The white dashed line in the AFM image marks the position of the cross section.

**Note 4. Properties of COC (TOPAS 8007)**

Chemical structure:

**
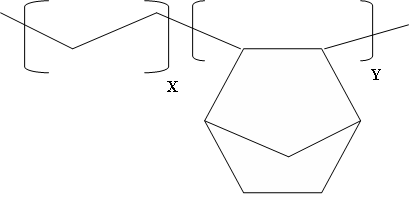
**

| Property | Value |
| --- | --- |
| COC/air interfacial tension | ~ 30 mN/m |
| COC/water interfacial tension | ~ 40 mN/m |
| Density | 1.02 g/cm^3^ |
| Tensile modulus | 2600 MPa |
| Water Absorption @24 h immersion at 23 °C | < 0.01% |

*The listed property comes from various sources. COC/air interfacial tension is from the website of TOPAS; and the COC/water interfacial tension is calculated from the measured water apparent angle; the density, tensile modulus, and water absorption come from the brochure of TOPAS COC.*

**Note 5. Demonstration of the thermodynamic stability of droplet on COC nanostructures**

Within 5 min of observation, the contact angle is almost unchanged (slight variation is caused by evaporation). In addition, the contact line of the droplet does not have any change for a long time. Therefore, it can be demonstrated that the droplet reaches a thermodynamically stable state in a short time at the beginning.


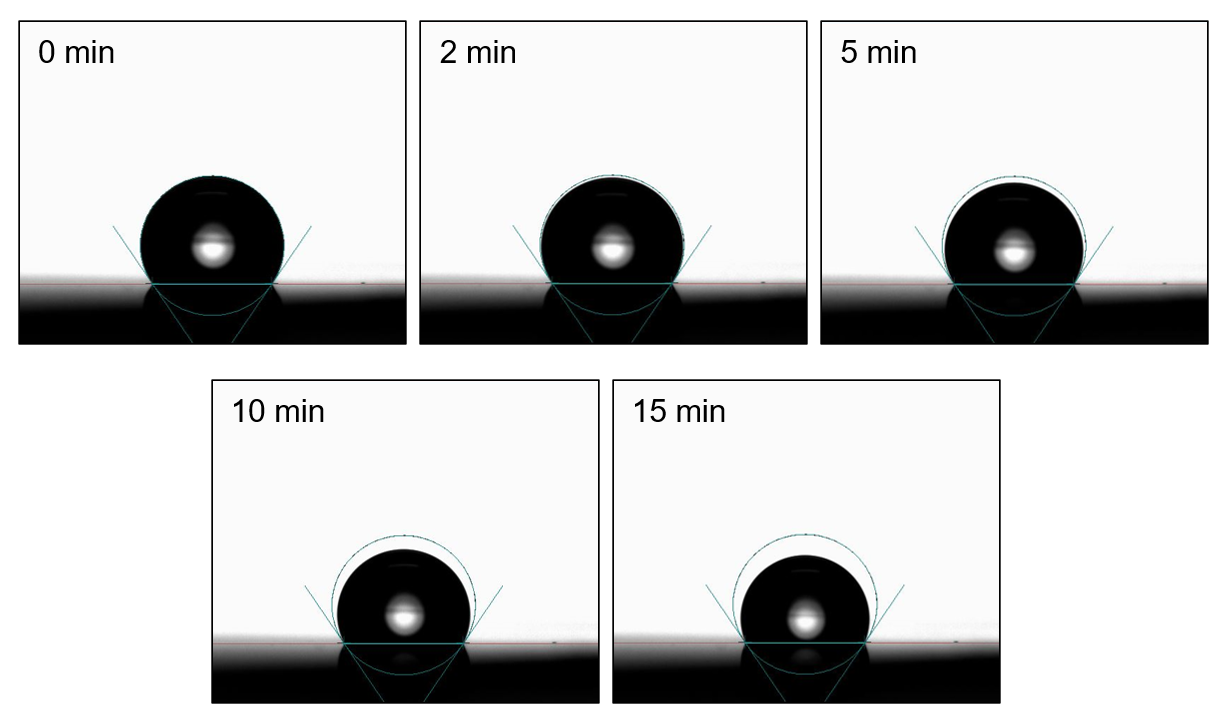


**Fig. S4:** Water droplet wetting behavior on the COC nanostructure from 0 min to 15 min.

**Note 6. Droplets jumping on the COC surface with gradient nanostructures**


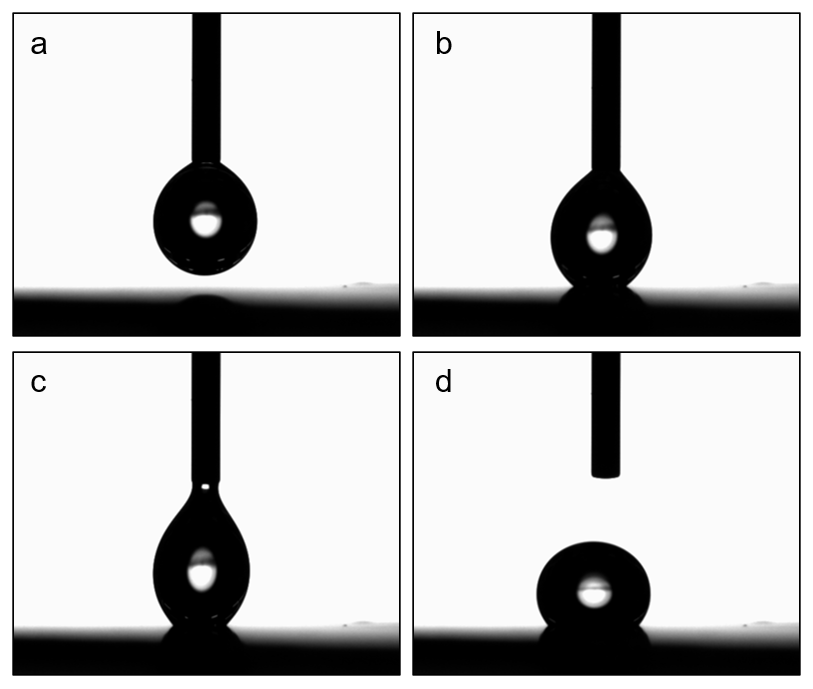


**Fig. S5**: Observation of the jumping of a water droplet deposited on COC surface with gradient nanostructures.

**Note 7. Detailed model derivation**

The apparent contact angle $\theta^{*}$ on a composite interfaces is determined by the liquid-vapor interface area ${(A}_{\mathrm{LV}})$, liquid-solid interface area${(A}_{\mathrm{LS}})$, projection area ${(A}_{P})$, and intrinsic contact angle $\theta_{e}$. If $f_{1}=A_{\mathrm{LS}}/A_{P}$ and $f_{2}=A_{\mathrm{LV}}/A_{P}$, as given by the Cassie-Baxter relation ^1^

$${\cos\theta}^{*}=f_{1}\cos\theta_{e} -f_{2}$$

In order to describe the nanostructure, three parameters are involved here: $a$ is the pattern feature size (diameter of pillars or holes), $p$ is the pattern period, and $H$ is the pattern depth.

(1) Wenzel state

$f_{1}=r$ ; $f_{2}=0$

then, ${\cos\theta}^{*}=r\cos\theta_{e}$

where $r=\frac{p^{2}+\pi aH}{p^{2}}$

(2) Cassie state

$f_{1}=f_{s}$ ; $f_{2}=1-f_{s}$

then ${\cos\theta}^{*}=f_{s} \left( 1+\cos\theta_{e} \right)-1$

where $f_{s}=\frac{{\pi a}^{2}}{{4p}^{2}}$ (as for pillars); $f_{s}=1-\frac{{\pi a}^{2}}{{4p}^{2}}$ (as for holes);

(3) Intermediate state 1

Only the pillar region (*R* = 0 mm to 6 mm) is taken into account.

$${\cos\theta}^{*}=f_{1}\cos\theta_{e} -f_{2}$$

where, *h* is defined as the immersion depth; $f_{1}=\frac{{\pi a}^{2}+4\pi ah}{4p^{2}}$ ; $f_{2}=\frac{4p^{2}-{\pi a}^{2}}{{4p}^{2}}$.

(4) Intermediate state 2

Only the pillar region (*R* = 0 mm to 6 mm) is taken into account.

$${\cos\theta}^{*}=f_{1}\cos\theta_{e} -f_{2}$$

where *x* is defined as the immersion ratio; $f_{1}=\frac{(1-x){\pi a}^{2}}{4p^{2}}+\frac{x(p^{2}+\pi aH)}{p^{2}}$; $f_{2}=\frac{(1-x)(4p^{2}-{\pi a}^{2})}{{4p}^{2}}$

The contact angles calculated from these four models are compared with the experimental results in Fig. 5.

**Note 8. Breakthrough pressure**


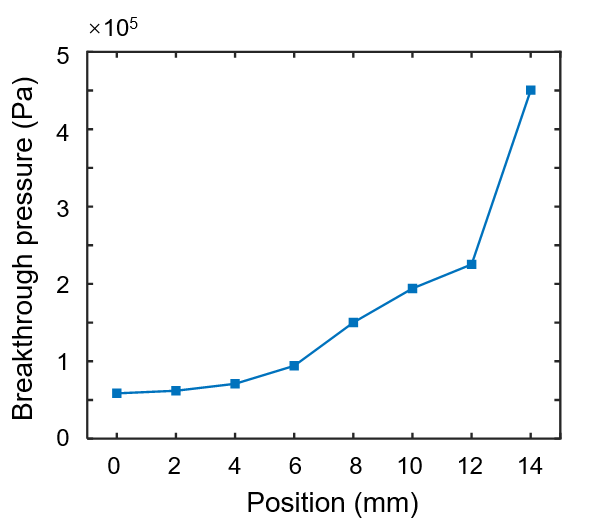


**Fig. S6:** Calculated breakthrough pressure at various positions of the COC surface with gradient nanostructures.

For the pillar region (*R* = 0 mm to 6 mm), the breakthrough pressure is given by

$$P_{b}={\frac{-4\pi a\gamma\cos\theta_{e}}{4p^{2}-{\pi a}^{2}}}$$

where $a$ is the diameter of pillars, $p$ is the pattern period, $\theta_{e}$is the intrinsic contact angle, and $\gamma$is the water surface tension.

For the hole region (*R* = 8 mm to 14 mm), the breakthrough pressure is given by

$$P_{b}={\frac{-4\gamma\cos\theta_{e}}{a}}$$

where $a$ is the diameter of holes; $p$, $\theta_{e}$, and $\gamma$are the same with the pillar equation.

**Note 9. Adhesive force**

The adhesive force is assessed by a high-sensitivity microelectromechanical balance system. The COC surface with gradient nanostructures is placed on the plate of the balance system. A 3.5 μL water droplet is suspended on a metal ring, and the structured COC film is brought into contact with the water droplet. Then the droplet is compressed on the structured COC film at a rate of 0.05 mm/s with a distance of 0.2 mm. Next, the structured COC film leaves the water droplet after contact. The balance force increases gradually and reaches its maximum at the end of the process. The maximum force at the position just before the water droplet leaves the surface is recoded as the adhesive force.


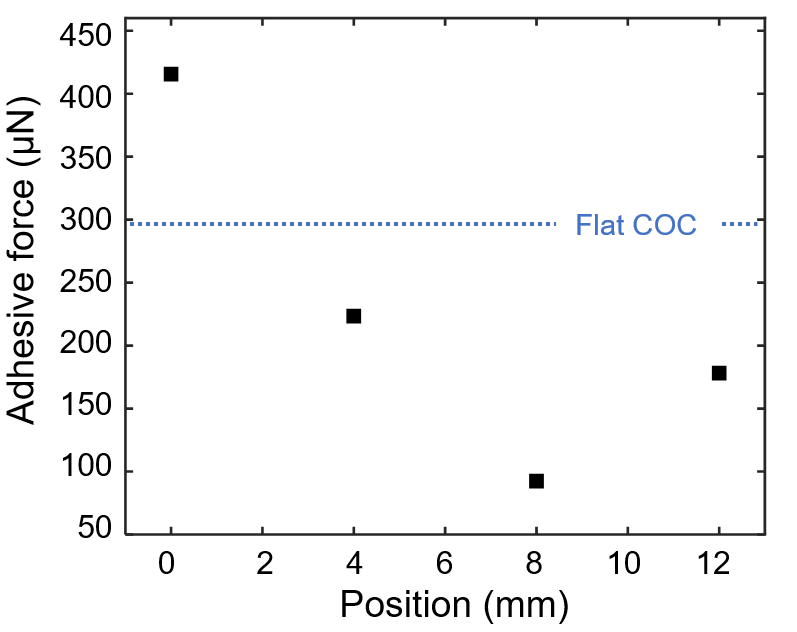


**Fig. S7:** Measured adhesive force on flat COC film and COC film with gradient nanostructures.

**Note 10. Squeezing test**


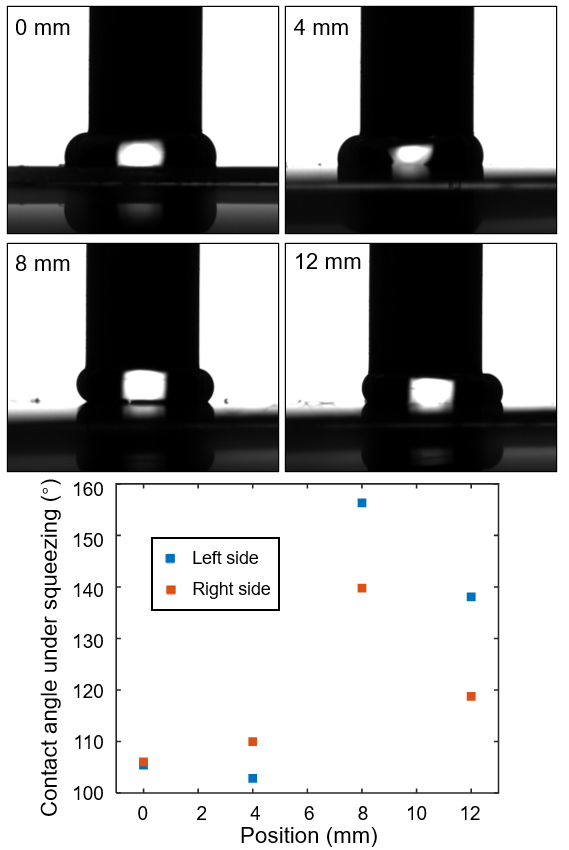


**Fig. S8:** Contact angle of the structured COC surface under squeezing.

**Note 11. Contact angle versus filling ratio**


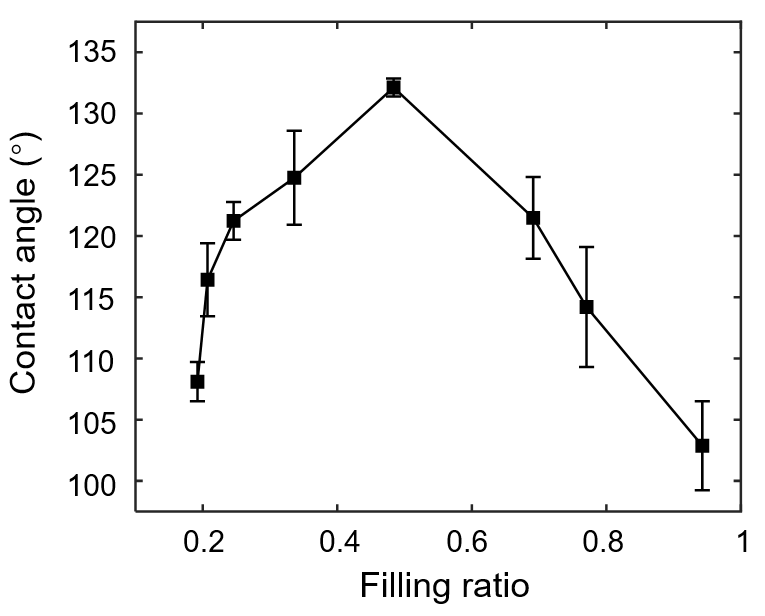


**Fig. S9:** The plot of water contact angle on COC nanostructures with various filling ratio.

**Note 12. Hypothesis verification**


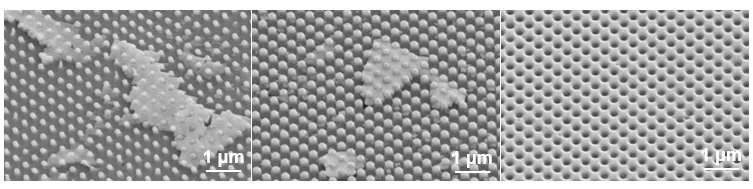


**Fig. S10:** SEM images of structured COC film after performing advancing and receding experiment with NaCl solution.

**Reference**

1 Cassie, A. & Baxter, S. Wettability of porous surfaces. *Transactions of the Faraday society* **40**, 546-551 (1944).
